# Editorial: The Effects of Enriched Environment on NK cells and Macrophages and Their Underlying Mechanisms

**DOI:** 10.3389/fimmu.2022.862488

**Published:** 2022-02-24

**Authors:** Yoshiro Kobayashi, Cristina Limatola, Yu Gan

**Affiliations:** ^1^ Toho University, Tokyo, Japan; ^2^ Instituto di Ricovero e Cura a Carattere Scientifico (IRCCS) Neuromed, Pozzilli, Italy; ^3^ Department of Physiology and Pharmacology, Laboratory Affiliated to Istituto, Pasteur Italia, Sapienza University, Rome, Italy; ^4^ State Key Laboratory of Oncogenes and Related Genes, Shanghai Cancer Institute, Renji Hospital, Shanghai Jiao Tong University School of Medicine, Shanghai, China

**Keywords:** enriched environment, NK cells, Macrophages, M1/M2, cytokine

There are two types of stress, namely distress and eustress. An enriched environment (EE) as a model of eustress has been studied mainly in the field of neuroscience, but EE has also recently been studied in the field of immunology and cancer research. Since EE affects various immune cells, including NK cells and macrophages, we called for papers to shed light on such cells and their interactions. Four review articles and two original papers were published. Here, we introduce each manuscript briefly and connect them.

Microglia, macrophages in the brain, may interact with NK cells, which often reside in a brain tumor. Xiao et al. describe the underlying mechanism of the effects of EE on NK cells in terms of cancer treatment and/or prevention. The group of Dr. L. Cao is the first to identify hypothalamic BDNF as a key regulatory molecule in EE. The group also investigated other molecules, which are regulated in the hypothalamus of EE mice, none of which can recapitulate the peripheral phenotypic changes induced by EE in the way that hypothalamic BDNF can. BDNF produced in the brain of EE mice then modulates NK cell activity through IL-15 expressed by microglia in the context of glioma. Although there are shared molecules, such as norepinephrine and glucocorticoid, between EE and distress, the degree of change in the molecules are different from each other, whereas BDNF is one of the key molecules unique to EE.

Recently, Mormino et al. modified microglia with recombinant adeno-associated virus serotype 2 (rAAV2) carrying IL-15 (rAAV2-IL-15) to force the production of IL-15. rAAV2-IL-15 microglia increased the NK cell viability in coculture. Intranasally delivered rAAV2-IL-15 microglia entered the brain and reached glioma. The microglia triggered the interplay with NK cells *in vivo*, enhancing NK cell recruitment and pro-inflammatory microglial phenotype (Arg1 negative, increased number of branches, coverage of a wider parenchymal region) in tumor mass of glioma-bearing mice, and ultimately counteracted tumor growth. Their finding on the shape change of microglia is interesting because of the review article by Carvalho-Paulo et al. on the analysis of the morphometric features of microglia. They also introduce the morphometric features of the microglia of mice infected with neurotropic viruses, such as Dengue virus and arbovirus, which were more complex than those of control mice.

An interaction of NK cells with macrophages is also seen in other sites than the brain, as reviewed by Russo et al. Although tumor associated macrophages (TAM) are usually M2-like, which inhibit NK cell activity, TAM can be converted to M1-like, for instance, by targeting a scavenger receptor MARCO, to produce IL-15 and then augment NK cell activity.

EE also affects other macrophages than microglia. As shown in the study by Sakama et al., irradiation caused DNA double strand break in the lungs, which was repaired afterward, while irradiation induced *TNF* gene expression in the lungs, presumably lung macrophages, which was then diminished. EE accelerated such DNA repair. The EE augmented *TNF* gene expression initially and anti-inflammatory marker *Arg1* gene expression later on. Although the serum leptin level in standard environment (SE) mice recovered after irradiation, the level in EE mice remained low even after irradiation, suggesting that EE maintains an anti-inflammatory status to suppress carcinogenesis.

A sedentary lifestyle, which is mimicked by the SE in mice, is known as one of the contributors to fatty liver disease. A combination of such a lifestyle with a high fat diet causes lipotoxicity in the liver, whereas a combination with a high intake of glucose and fructose causes glucotoxicity in the liver. Such liver injury ultimately causes fatty liver disease. Xu et al. described the underlying mechanism of fatty liver disease, focusing on the principal role of hepatic macrophages. Kupffer cells act as key players in the progression of fatty liver disease through phenotype switch from M2 to M1 and inflammatory cytokine production.

Overall, each paper contributes to research in this field and will contribute to future studies that widen our knowledge on the effects of EE and serve in developing clinical applications.

## Author Contributions

YK wrote the editorial, while CL and YG modified the editorial. All authors contributed to the article and approved the submitted version.

## Conflict of Interest

The authors declare that the research was conducted in the absence of any commercial or financial relationships that could be construed as a potential conflict of interest.

## Publisher’s Note

All claims expressed in this article are solely those of the authors and do not necessarily represent those of their affiliated organizations, or those of the publisher, the editors and the reviewers. Any product that may be evaluated in this article, or claim that may be made by its manufacturer, is not guaranteed or endorsed by the publisher.

